# Medical Ethics

**Published:** 1852-11

**Authors:** 


*Medical Ethics.*—The perusal of an article in the *London Medical Times*, touching a formidable feud which has recently sprung up in the good city of Edinburgh, forcibly reminds us of certain sins too common in our profession. The offense to which we allude is the too common practice of *criticising* the professional conduct of our cotemporaries. Examples, unhappily, of such misdeeds are too common everywhere, and we might readily find sufficient illustrations in our own country; but as it is no very pleasant duty to review our neighbor's conduct, and finding pertinent cases abroad, we prefer, on the present occasion, to cite a single instance of the latter. Moreover, as it is not our design to write a homily on medical ethics, we will mention only a single case, which, from its remoteness of location, can excite no personal ill feeling.

Several foreign medical journals have recently made allusions and contained articles in relation to an acrimonious personal controversy between Professors Simpson and Miller, of Edinburgh. It appears that the former performed some sort of surgical operation (the character not stated, but probably *vaginal*) on the wife of a medical gentleman. The patient died, *suddenly*, two days after the operation. An impression was at once produced that the patient died of hæmorrhage, but neither Prof. Simpson, nor Prof. Christison (who was called in consultation) detected the hæmorrhage. Many rumors were immediately set afloat in regard to the case, all more or less censuring the operator. A gossiping Doctor of the town, happening to have business with an upholsterer, was told by the latter that the mattress on which the patient lay had been sent to him to be cleaned, and that it was stained with blood. The gossiping Doctor immediately communicated this delectable morceau of intelligence to Professor Miller, who in turn mentioned it to Professor Henderson, the *homœopathist* who disgraces the University of Edinburgh. Nothing further was necessary to give the report currency. The homœopathist did not fail to circulate the statement, that a patient of Dr. Simpson's had been suffered to die of hæmorrhage, after an unimportant operation. Dr. Henderson's author being inquired for, the report was at once traced back to Mr. Miller. Dr. Simpson, as a matter of course, felt greatly aggrieved that his colleague, Mr. Miller, should be instrumental in circulating such a report, and, as it appears, an unfounded one. Of Dr. Henderson nothing better could be expected. The facts of the case show that the stain on the mattress, after all, was quite small, about such as would follow an ordinary operation, but not sufficient to cause death. Dr. Christison was of opinion that the patient died of low peritonitis.

Dr. Simpson, in a communication to Dr. Christison, complained of the conduct of Mr. Miller, and thus the quarrel began, which seems likely to have no end. Mr. Miller, in defense of his conduct, alleges that the story of the bloody mattress came to him incidentally, and that it was casually mentioned to Dr. Henderson, without any sinister motive.

It cannot be doubted that the conduct of Mr. Miller was in every sense wrong and unprofessional, whatever the motive. In the first place it was wrong to give currency to a report derogatory to a member of the profession, (and that member his *own colleague*,) on the mere statement of an unprofessional man, altogether unused to judge of such matters; and, even if *true*, it did not become his colleague to circulate the story; but, above all, it was inexcusable to make such a statement to an avowed *homœopathist*, although he, too, was a colleague. We can readily admit that Mr. Miller's remarks were uttered in carelessness rather than from sinister motives; but it is just such carelessness, (or recklessness more properly) which so often strikes at a man's reputation, under the shield of *inadvertence!*

In relation to the quarrel which has so unfortunately disturbed the harmony of the Edinburgh profession, the *London Medical Times* holds the following language:

"This dispute touches a vital doctrine of medical ethics; a doctrine admitted by all, but practiced by few. The first law of courtesy and professional honor is, that one medical man shall not discuss, criticise, much less condemn the conduct of another."

This doctrine is worthy of all commendation, and without it the profession becomes a disjointed assemblage of moral assassins. Gross blunders, or obvious malpraxis, growing out of absolute ignorance, are not subject to such restrictions, but, on the contrary, deserve unlimited condemnation. If a surgeon, through ignorance of anatomy, destroy his patient by wounding a vital part; or, if a physician from gross incapacity, should administer corrosive sublimate in *ten grain* doses, and thus destroy life, no stringency of ethics can require silence, but all such acts deserve, and will receive universal condemnation. But the case is quite different when a mere *accidental* injury is inflicted; thus, the most skillful surgeon may accidentally wound the intestine in operating for strangulated hernia, and the most competent physician by administering a grain of opium in a case not precisely adapted to its action, may induce fatal cerebral congestion. These and similar accidents are the results of errors of judgment, or sheer casualties, which demand the sympathy rather than the censure of the more fortunate of the profession.

There are three classes of persons who injure their brethren by untimely criticisms: first, those who do it inadvertently and without improper motive: second, a class who indulge in criticism from a mere love of gossip, without a *desire* to injure the person who becomes the subject of remark; and, third, a class of wicked revilers who love detraction for the deed itself, and gloat over the defects, real or imaginary, of their fellow beings. These three classes exhibit widely different degrees of censurableness: the inadvertent man is censurable for his recklessness of the interests of his brother; the conduct of the gossiping man is reprehensible because he loves his neighbor's character less than his own gratification; and the malignant reviler deserves the severest condemnation for his wicked propensity.

How different would be the condition of the medical profession, if these criticisers could be induced to pause and *think* before speaking. It is a much more genial office to praise than censure a co-laborer; and the same keenness of optics guided by a judicious impulse, would find more to commend than disparage in the conduct of their brethren. And then the effects on the whole profession would be highly beneficial. We should, with this liberal course, present an unbroken front, which would appall the swarms of empirics, and command the entire confidence of the community.

Let us then be more guarded, and less censorious, and let us direct our efforts to the development of science, rather than the destruction of individual character.— *Western Lancet, Editorial.*

